# Compounded glycopyrrolate is a compelling choice for drooling children: five years of facility experience

**DOI:** 10.1186/s13052-021-01173-7

**Published:** 2021-11-06

**Authors:** Davide Zanon, Cristina Tumminelli, Anna Maria Chiara Galimberti, Lucio Torelli, Alessandra Maestro, Egidio Barbi, Natalia Maximova

**Affiliations:** 1 Pharmacy and Pharmacology Unit, Institute for Maternal and Child Health, IRCCS Burlo Garofolo, via dell’Istria 65/1, 34137 Trieste, Italy; 2 Department of Medicine, Surgery and Health Sciences, University of Trieste, Piazzale Europa 1, 34127 Trieste, Italy

**Keywords:** Drooling, Sialorrhea, Galenic, Compounded drug, Glycopyrrolate

## Abstract

**Background:**

Describe the efficacy of a galenic glycopyrrolate formulation and its impact on patients with sialorrhea Quality of Life (QoL), including costs analysis.

**Methods:**

We performed a retrospective observational study on 21 patients who received a custom-formulated galenic glycopyrrolate syrup for sialorrhea for an average period of 14.3 months. We analyzed the telephone interviews with elaborated and validated questionnaires and the therapy costs comparing the brand marketed drug with the galenic formulation.

**Results:**

Overall, 16 out of 21 patients (76.2%) reported a significant improvement in sialorrhea and QoL. In 14 subjects (66.7%), there was a remarkable decrease in the drooling severity; 10 individuals (47.6%) reported a reduction in drooling frequency. Nine patients experienced at least one adverse effect of glycopyrrolate therapy, and three of them stopped the treatment. No severe side effects were observed. The galenic drug significantly reduced costs for patients.

**Conclusions:**

An oral glycopyrrolate solution easily administered to children with brain injuries is not commercially available in many European countries. This study demonstrates the efficacy of a compounded glycopyrrolate syrup on drooling severity, frequency and ensures a better QoL in patients and their caregivers.

## Background

Sialorrhea (drooling) is a common, unpleasant, and frequently debilitating problem for pediatric patients who suffer from brain injuries (cerebral palsy, severe intellectual disability, encephalopathies, and encephalitis), muscular/neuromuscular disorders (amyotrophic lateral sclerosis, facial paralysis), and other conditions that impair the physiology of deglutition [1, 2]. Remarkably, around 35% of children with cerebral palsy have problematic drooling [3]. Sialorrhea results from impaired coordination in the swallowing process, with a low saliva clearance and consequent saliva leakage from the oral cavity. Patients with chronic sialorrhea are at risk of developing respiratory complications (aspiration pneumonia, chronic cough, suffocation) and skin lesions (irritative dermatitis, cheilitis, perioral fissures). The condition has a significant impact on the QoL [4], especially if one considers the already complicated daily routine for patients and their caregivers. Several therapeutic strategies are available: behavioral interventions [5, 6], drug therapies, such as botulinum toxin, glycopyrrolate, scopolamine, and benztropine; and surgical treatment (gland removal, duct ligation, or neurectomy surgery) [7, 8]. Glycopyrrolate is an anticholinergic drug that blocks the muscarinic acetylcholine receptors, M1, and M3, in the salivary glands [9]. Formulations with different half-lives exist.

Glycopyrrolate oral solution is usually prescribed initially at 0.02 mg/kg three times daily and titrated in 0.02 mg/kg increments every 5–7 days, based on therapeutic response and adverse reactions. The maximum recommended dose is 0.1 mg/kg three times a day, not to exceed 1.5–3 mg per dose based on weight. Glycopyrrolate is recommended to be administered at least 1 h before or 2 h after meals [10].

The oral bioavailability of glycopyrrolate varies widely; it has a rapid plasma distribution and is eliminated by renal excretion [11]. The lack of a selective salivary-gland action explains the incidence of anticholinergic adverse effects such as xerostomia, tachycardia, irritability, constipation, and urinary retention. Compared to atropine and scopolamine, glycopyrrolate penetrates the blood-brain barrier in smaller amounts, limiting the central nervous system’s adverse effects.

The most common adverse reactions, with an incidence higher than 30%, are dry mouth, vomiting, constipation, flushing, and nasal congestion. Constipation or intestinal pseudo-obstruction may present with abdominal distention, pain, nausea, or vomiting. Dosage adjustment and patient assessment are required for constipation, particularly within 4–5 days of initial administration or after a dose increase. Care should be taken in the presence of renal impairment.

Although the drug’s efficacy on the drooling severity and frequency has already been described, limited evidence is available on its impact on the patients’ QoL and their caregivers. Notably, no glycopyrrolate syrup is commercially available in Italy to facilitate oral administration both by mouth or by gastrostomy, so it must be imported from other countries where it can be purchased [12].

This study aims to measure the clinical safety and effectiveness of a galenic glycopyrrolate liquid formulation in pediatric patients with sialorrhea, to evaluate whether glycopyrrolate’s use leads to improved patients’, their caregivers’ QoL, and to analyze the impact on costs.

## Methods

### Study design

This is a retrospective observational study on a group of pediatric patients treated for sialorrhea at the Pediatric Children Hospital of Trieste (Italy) between March 2015 and December 2019. The inclusion criterion was to be a pediatric patient undergoing treatment with galenic glycopyrrolate for sialorrhea. The exclusion criteria were language difficulties and lack of consent from the patient or caregiver.

Approval was obtained from the Internal Review Board for retrospective data collection (final reference no. 12/17), and, where required, parental consent to interview patients and caregivers and collect anonymous data.

### Procedures

The galenic glycopyrrolate formulation was produced by the Pediatric Institute Pharmacy and was prescribed at a dose of 0.021 mg/kg. Glycopyrronium bromide solution was compounded using a phosphate buffer to obtain a syrup with a range of pH around 5.6. The final concentration of the active substance was 0.5 mg/ml. The low drug concentration permitted the syrup to remain sweet for oral administration [13, 14].

### Outcome measures

All patients undergoing treatment with galenic glycopyrrolate for sialorrhea or their parents were contacted by telephone and invited to attend an interview. During the conversation, we collected the following clinical data: primary pathology, comorbidities, past medical therapies, surgical interventions, such as Nissen’s fundoplication, tracheostomy or percutaneous endoscopic gastrostomy, and concurrent drug administration.

Two specific questionnaires were administered to compare the clinical severity of drooling and the relative impact on the QoL before starting the therapy (T0) and after the therapy (T1).

**Fig. 1:**
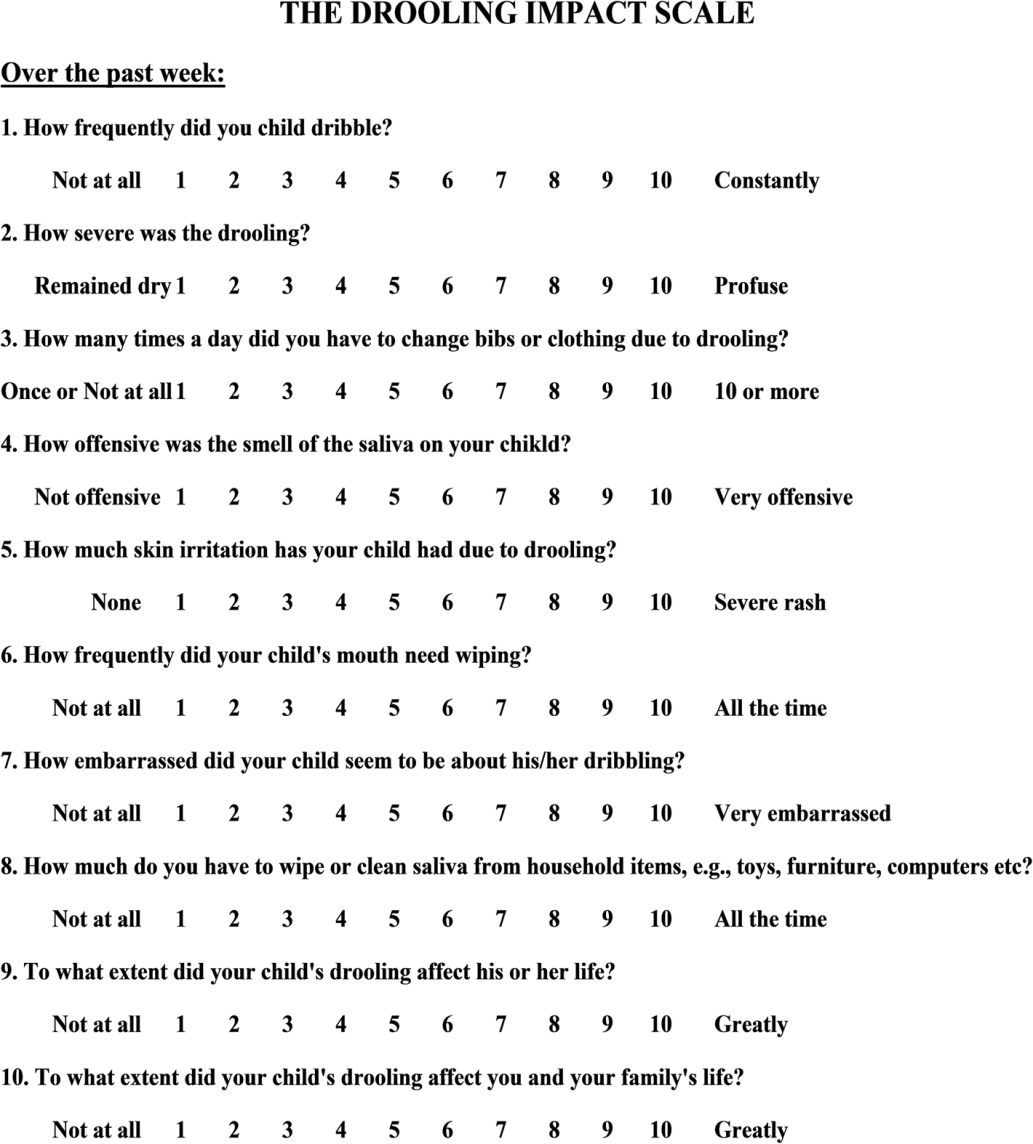
Drooling Impact Scale^13^

**Fig. 2:**
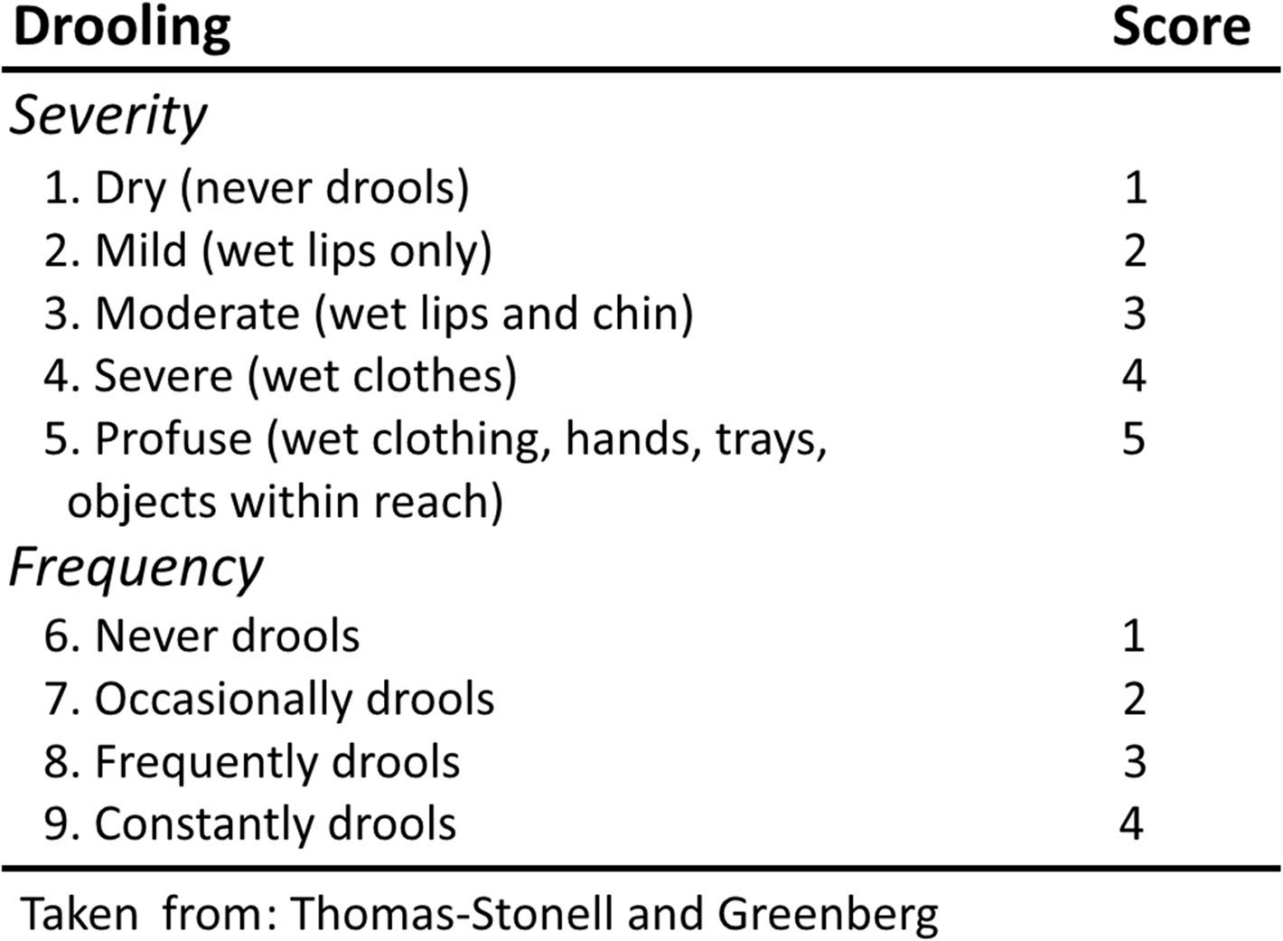
Thomas-Stonell and Greenberg Drooling Rating Scale

The first questionnaire was the “Drooling Impact Scale” (Fig. 1), a standardized tool consisting of ten questions relating to sialorrhea, its daily management, and its influence on the child’s psycho-physical well-being [15]. The second questionnaire was the “Drooling Severity and Frequency Scale” aimed at obtaining an objective assessment of the severity and frequency of salivation (Fig. 2) [16].

### Statistical analysis

For the data’s statistical analysis, differences between groups or between T0 and T1, we employed non-parametric statistical Mann-Whitney and Wilcoxon tests. The *p*-value was considered significant if *p* < 0.05.

### Costs evaluation

We evaluated three different price quotations by comparing them to the price of galenic glycopyrrolate.

## Results

**Fig. 3:**
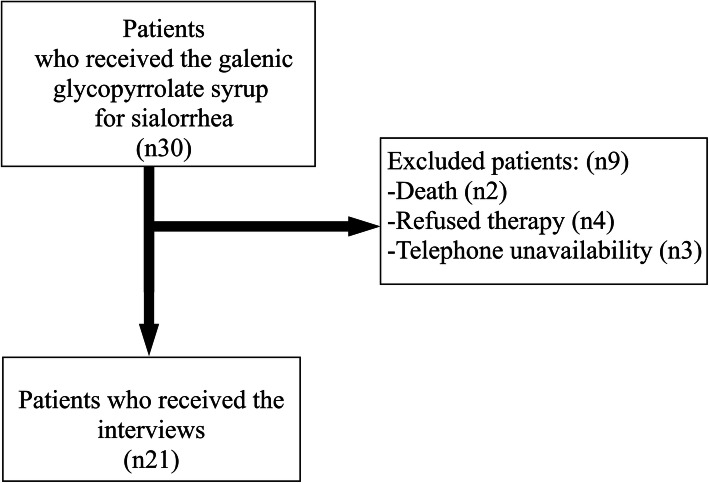
flow chart of enrolled patients

Thirty patients (18 males and 12 females) aged 2 to 19 years (median 12 range 2–19) were recruited based on all the galenic glycopyrrolate prescriptions to treat sialorrhea provided by the internal pharmaceutical database of the Burlo Garofolo Pediatric Institute (Fig. 3).

The final population consisted of twenty-one pediatric patients between three and twenty; the study group’s demographic data are shown in Table 1. They all suffered from drooling secondary to various diseases. The average length of treatment was 14.3 months (SD 13.4 months), with a minimum duration of 2 weeks (0.5 months) and a maximum of 36 months.

**Table 1:** Features of the Population under Study

Number of patients (%)	21 (100)
Sex, number (%)	
Male	14 (66.7)
Female	7 (33.3)
Age (median range)	12 (2–19)
Primary pathology, number (%)	
Cerebral palsy	7 (33,3)
Encephalopathy	5 (23,8)
Suprabulbar paralysis	1 (4,8)
SMA	2 (9,5)
Herpetic encephalitis	1 (4,8)
Generalized dyspraxia	1 (4,8)
Anorexia	1 (4,8)
Genetic syndromes	3 (14,3)
Gross Motor Function Classification System, number (%)	
GMFCS I	0 (0)
GMFCS II	2 (9,5)
GMFCS III	2 (9,5)
GMFCS IV	6 (23,9)
GMFCS V	11 (57,1)
PPI administration, number (%)	
Yes	13 (61,9)
No	8 (39,1)
PEG, number (%)	
Yes	10 (47,6)
No	11 (52,4)
Fundoplication, number (%)	
Yes	6 (28,6)
No	15 (71,4)
Tracheostomy, number (%)	
Yes	4 (19)
No	17 (81)
Current scopolamine therapy, number (%)	
Yes	2 (9,5)
No	19 (90,5)
Antiepileptic treatment, number (%)	
Yes	13 (61,9)
No	8 (39,1)
Other drugs, number (%)	
Yes	15 (71,4)
No	6 (28,6)
Past therapies for drooling, number (%)	
Scopolamine	3 (14,3)
Botulinum toxin	5 (23,8)

*PPI* Proton pump inhibitors, *PEG* Percutaneous endoscopic gastrostomy

**Fig. 4:**
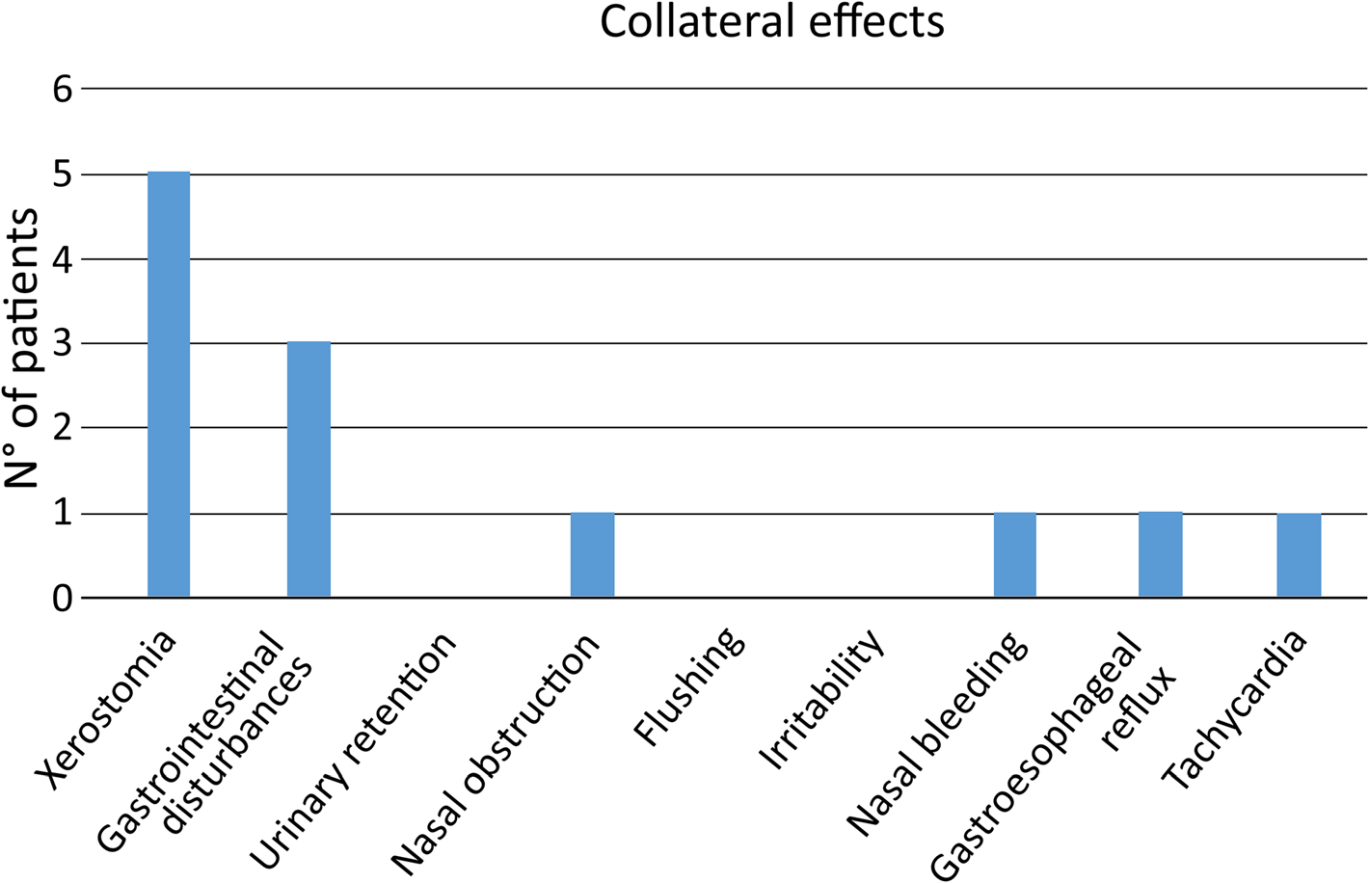
Frequency of Adverse Effects of Galenic Glycopyrrolate Treatment

We observed that 42.9% of the subjects (9/21) reported at least one adverse effect regarding clinical safety. We recorded xerostomia in five patients, gastrointestinal symptoms (constipation/diarrhea/vomit) in three, nasal obstruction in one, gastroesophageal reflux in one, nasal bleeding in one, and tachycardia in another one. Three patients out of nine experiencing side effects decided to stop the therapy. No patient showed adverse effects (Fig. 4).

**Fig. 5:**
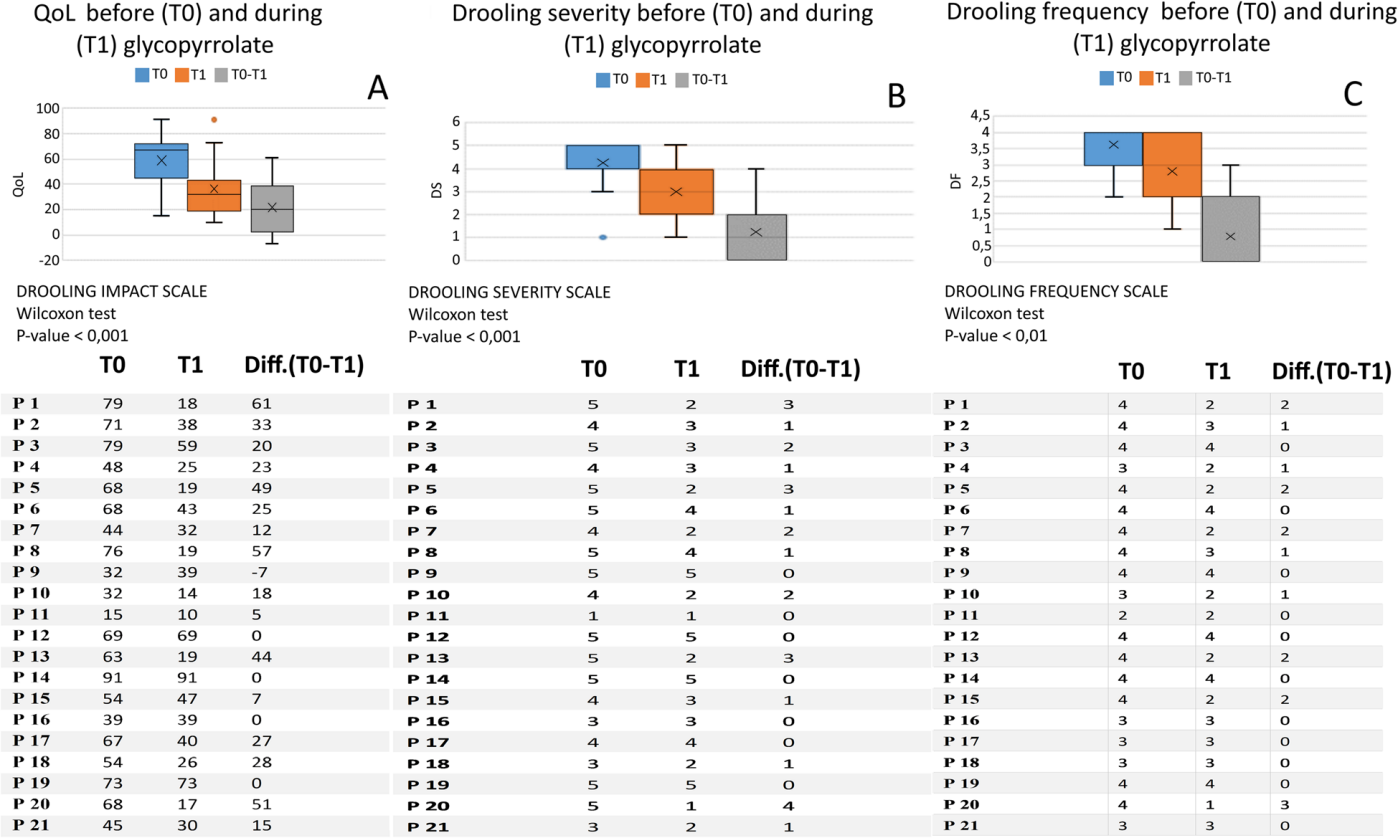
Effectiveness of Galenic Glycopyrrolate Treatment. Significant improvement in the QoL of patients and caregivers (*p* < 0.001), and reduction in severity (*p <* 0.001) and frequency of drooling (*p* = 0.01)

The drooling impact scale revealed statistical differences between T0 and T1 (Wilcoxon: *p* < 0,001) (Fig. 5A): in particular, 16 out of the 21 patient caregivers (76.2%) reported an improvement in sialorrhea, their QoL, and in that of their children. There were no changes in 4 out of 21 cases; one patient complained of a worsening in the sialorrhea despite treatment. Results from the Drooling Severity and Frequency Scale analysis showed a statistical disparity between T0 and T1 (Wilcoxon: *p* < 0.001) too (Fig. 5B): in fourteen patients (66.7%), there was an improvement in the severity of the drooling, while the other seven remained stable. No one described worsening of Drooling Severity (DS) with glycopyrrolate. Regarding the Drooling Frequency (DF) (Fig. 5C), a statistical difference between T0 and T1 (Wilcoxon: *p* < 0.01) was observed: ten patients (47.6%) reported a positive change, and in eleven patients, the DF was stable under glycopyrrolate treatment. No patient complained of deterioration of DF.

### Duration of therapy

We divided the patients according to the length of treatment to evaluate how this could affect the glycopyrrolate efficacy: short duration (0.5–12 months, 13 patients), intermediate duration (13–24 months, 3 patients), and long duration (25–36 months, 5 patients). The most evident improvement was described by a single patient who took glycopyrrolate for 24 months; the subjects who received the drug for 25–36 months had a 1 or 2 points change in drooling frequency, while there was no significant improvement if the treatment had been carried out for less than 12 months. As for QoL, all patients in the second (13–24 months) and third (25–36 months) group profited from glycopyrrolate therapy, with the maximum benefit reported within the 25–36 months group, while the patients who described no improvements were in the short-duration group (0.5–12 months).

### Methods and frequency of administration

Twelve patients (57.1%) took glycopyrrolate orally, the other nine via percutaneous endoscopic gastrostomy (PEG). We compared the improvements in DS, DF, and QoL described in patients receiving oral glycopyrrolate versus those who took it via PEG. The *p*-value was not statistically significant in any of the three analyses.

Nine patients received glycopyrrolate twice a day, eleven subjects three times a day, and only one individual took it four times a day. There was no statistically significant improvement on the DS, DF, and QoL depending on the administration’s frequency.

### Gastroesophageal reflux

We considered two groups separately: 14 patients (66.7%) with gastroesophageal reflux disease (GERD), treated with proton pump inhibitors or undergoing Nissen surgery, and seven children without GERD. We did not find any significant difference between the two groups concerning DF (Mann-Whitney: p = NS), DS (Mann-Whitney: p = NS), and QoL (Mann-Whitney: p = NS). These data revealed that glycopyrrolate improved the drooling severity equally in children with or without GERD.

### Administration and meals

Fourteen patients received glycopyrrolate on an empty stomach, while the other seven took it near meals. The Mann-Whitney test proved no statistically significant correlation between DS, DF, QoL, and drug administration concerning meals.

Although this administration schedule is in contrast to what is known about glycopyrrolate, the absorption of which is generally influenced by high-fat food, so the drug should be administered at least 1 h before or 2 h after a meal, it is in line with the package leaflet of the commercial product.

### Prices, costs-saving, and drug accessibility

Glycopyrrolate is not marketed in Italy and is not dispensed by the Italian National Health System. The imported oral suspension price (1 mg/5 ml 473 ml) is around 530 USD [17]. The cost of the galenic compounded glycopyrrolate (1 mg/2 ml 190 ml), compared to brand drug, in the same form and the same total drug quantity, is around 32,83 euros (consisting of the price of the substances is 6,46 euros, pharmacist’s honorary 23,45 euros according to the Italian law, a supplement of 2,50 and bottle price of 0,42 euros). The mean cost saving is around 581 euros per bottle.

## Discussion

This study shows that a galenic glycopyrrolate solution significantly improves drooling severity and patients’ and caregivers’ QoL with reduced costs.

Sialorrhea is a severe issue in the daily life of children with debilitating conditions [18]. Drooling affects up to 37.5% of children with cerebral palsy [19], and this percentage is even higher among those who attend special daycare centers: 58% reported drooling, and 33% drooled severely [20]. The sialorrhea treatment aims to improve the QoL for both the patient and caregivers [21]. In this study, we wanted to evaluate the effectiveness of a galenic glycopyrrolate syrup on sialorrhea and improve the QoL related to the treatment. More than half of our population (52.4%) described the baseline sialorrhea as profuse, and 66.6% reported frequently drooling before therapy. To evaluate the efficacy and the impact on the QoL, we submitted specific questionnaires to the patients’ caregivers. The data derived from the subjective “Drooling Impact Scale” showed that 76.2% of the interviewees stated a significant improvement in their children’s and families’ QoL. A positive impact of the glycopyrrolate on the sialorrhea severity and frequency also emerged from the objective “Drooling Frequency and Severity Scale.”

These results were consistent with previous studies, demonstrating a decrease in drooling’s severity and frequency with limited side effects [22, 23].

Glycopyrrolate can lead to major side effects, such as tachycardia and urinary retention, and minor adverse effects such as xerostomia, flushing, irritability, gastrointestinal problems, and nasal bleeding [23–25]. In our series, the glycopyrrolate appeared to be substantially safe, although adverse effects were reported by 42.9% of the patients. Indeed, only one patient had tachycardia, while the remaining adverse effects were considered minor.

No worsening of GERD was reported after Glycopyrrolate administration, and none of our patients mentioned irritability. The 33.3% of caregivers and patients with adverse effects stopped the therapy, while the others considered these symptoms to be manageable and continued to consume glycopyrrolate. Remarkably 77.7% of those who experienced adverse effects stated that glycopyrrolate improved their QoL.

The glycopyrrolate oral solution is not available in Italy, and its import from other countries is inconvenient for families and involves a significant economic burden. The galenic glycopyrrolate formulation makes the therapy accessible to a higher number of children who need it. Furthermore, this study allowed us to obtain data on glycopyrrolate safety in a solution taken for a more extended period than the data available in the literature, which appeared relevant only to short-term intermittent use [26].

These outcomes led us to highlight some variables that could affect the drug’s effects. We saw that the longer the therapy was administered, the greater were the reduction in drooling frequency and the QoL improvement.

The literature often considered a three-dose daily regimen appropriate regarding posology; however, only 52.4% of our patients took the drug three times a day, while the remaining received it twice. Interestingly, there was no difference in glycopyrrolate’s effect depending on the posology. The possibility of not giving the child an additional dose of glycopyrrolate could lead to greater compliance, especially in those who attended school or participated in other activities. We found no differences between taking the drug on an empty or a full stomach or between oral or PEG-button administration. A 2012 study demonstrated that 30% or more participants developed adverse effects when they increased the drug doses [11]. Our study did not investigate whether a dose increase led to a concomitant increment in adverse effects; however, this is undoubtedly an aspect that would certainly be worth pursuing in future studies. This study’s limits are its retrospective nature, the possible memory biases, and patients’ small sample. Points of strength are the length of follow-up and the investigation through validated QoL questionnaires.

Finally, since glycopyrrolate is a non-priority drug for the Italian National Health System, its cost should be covered by the local Health Authority or by parents. This study highlights a significant gap between the price of the imported, marketed drug and the compounded drug, but it shows no substantial differences in efficacy than the available studies using the industrial product.

In conclusion, we proved that a galenic glycopyrrolate solution provides a significant improvement in drooling severity and patients’ and caregivers’ QoL. Adverse effects were reported in 42.9% of this population but were never severe and led to treatment interruption only in a minority of patients.

## Data Availability

Not applicable.
